# Effect of Surfactant Molecular Structure on Emulsion Stability Investigated by Interfacial Dilatational Rheology

**DOI:** 10.3390/polym13071127

**Published:** 2021-04-02

**Authors:** Yuejie Jin, Dingrong Liu, Jinhua Hu

**Affiliations:** 1 State Key Laboratory of Food Science and Technology, Jiangnan University, Wuxi 214122, China; j879954463@gmail.com; 2 School of Food Science and Technology, Jiangnan University, Wuxi 214122, China; liudingrong1998@163.com; 3 Synergetic Innovation Center of Food Safety and Nutrition, Jiangnan University, Wuxi 214122, China

**Keywords:** interfacial dilatational rheology, dynamic interfacial tension, interfacial viscoelasticity, water-in-oil emulsion, emulsion stability

## Abstract

Polyglycerol polyricinolate (PGPR) and polyglycerol-2 dioleate were selected as model surfactants to construct water-in-oil (W/O) emulsions, and the effect of interfacial rheological properties of surfactant film on the stability of emulsions were investigated based on the interfacial dilatational rheological method. The hydrophobicity chain of PGPR is polyricinic acid condensed from ricinic acid, and that of polyglycerol-2 dioleate is oleic acid. Their dynamic interfacial tensions in 15 cycles of interfacial compression-expansion were determined. The interfacial dilatational viscoelasticity was analyzed by amplitude scanning in the range of 1–28% amplitude and frequency sweep in the range of 5–45 mHz under 2% amplitude. It was found that PGPR could quickly reach adsorption equilibrium and form interfacial film with higher interfacial dilatational viscoelastic modulus to resist the deformation of interfacial film caused by emulsion coalescence, due to its branched chain structure and longer hydrophobic chain, and the emulsion thus presented good stability. However, polyglycerol-2 dioleate with a straight chain structure had lower interfacial tension, and it failed to resist the interfacial disturbance caused by coalescence because of its lower interfacial dilatational viscoelastic modulus, and thus the emulsion was unstable. This study reveals profound understanding of the influence of branched structure of PGPR hydrophobic chain on the interfacial film properties and the emulsion stability, providing experimental reference and theoretical guidance for future design or improvement of surfactant.

## 1. Introduction

Polymeric surfactants are now used to control the emulsion rheology and applied in various industries like oil recovery [1,2], cosmetics [3] and drug delivery [4]. Generally, the stability of emulsion limits its application. The emulsion instability is usually caused by emulsion flocculation, coalescence, Ostwald ripening, creaming, sedimentation and phase inversion. Emulsion destabilization occurs more frequently for those water-in-oil (W/O) emulsions such as margarine [5], sunscreen [6], since the low electrical conductivity of the continuous phase cause only steric forces are expected to stabilize the emulsions [7].In order to prepare stable W/O emulsions, the newly formed emulsion droplets are need to be protected by surfactants from these instability factors. The ideal surfactant requires short interfacial adsorption duration, strong ability to reducing interfacial tension, and high coverage over water-oil interfacial after adsorption [8,9]. The rapid adsorption of surfactant helps the interfacial tension reach a lower equilibrium value speedily to promote the droplets formation during emulsifying. A high coverage interfacial film on the surface of dispersed droplets formed by the surfactant can prevent the coalescence of emulsion droplets.

The interfacial properties of surfactant are mainly determined by its molecular structure [10]. Therefore, the investigation of effects of the molecular structure of surfactant on emulsion stability, especially from the perspective of molecular structure affecting the interfacial film properties, is beneficial to design and select surfactant properly and reasonably. For example, emulsion coalescence is related to the thinning and rupturing of interfacial liquid film between emulsion droplets. The fluid mechanics of droplet coalescence is essentially determined by the interfacial rheological properties of droplet film, particularly affected by its dilatational viscoelasticity [11,12,13], where the viscoelastic interface with high interfacial dilatation viscoelasticity facilitates the inhibition of flocculating droplets coalescence and resists the deformation of liquid film due to the mechanical disturbance [14]. In addition, the Ostwald ripening process, although mainly drove by the solubility of dispersed liquid, is also affected by surfactant and the rheological properties of droplet interface [15]. The compression−expansion of surfactant layers is slow in Ostwald ripening, so low frequency oscillation can be applied to investigate the response of droplet interface to the Ostwald ripening of emulsion. Above all, the dynamic interfacial tension and interfacial dilatation rheology are feasible methods to characterize and explore the mechanical properties of emulsion droplet interface film, including the deformation of interface film upon the mechanical disturbance during emulsification, flocculation, coalescence and Ostwald ripening, which are essentially important to illustrate the instability mechanism of emulsion.

The dynamic interfacial tension and interfacial dilation rheological properties of surfactants can be studied via the interfacial dilatation rheometer based on the Langmuir trough method. To investigate the macroscopic deformation [16] of droplet interface, the barriers expand or compress the interface at a constant rate or repeatedly, and subsequently the interfacial tension-area (π-A) isotherm is recorded to analyze the dynamic interfacial tension and interfacial adsorption characteristics. The interfacial dilatational rheology [17] characterizes a series of sinusoidal waves parallel to the interface and measures the interfacial dilatational viscoelasticity during oscillation, analyzing the resistance of interfacial film to mechanical disturbance. The change of amplitude or oscillation frequency of barriers are analogous to producing different mechanical disturbances. For details, the change of interfacial area caused by emulsion droplet coalescence can be studied by amplitude sweep [18], and the effects of different amplitudes on interface dilatational viscoelasticity help to explore and clarify the influence of interfacial film properties on the resistance to droplet coalescence. On the other hand, the compression-expansion changes of interface film during aging can be reflected by frequency sweep (within 0.2 Hz), and the analysis of effects of different frequencies on the interfacial dilatational viscoelasticity benefits to illustrate how the interfacial film properties resisting the emulsion Ostwald ripening [19]. The microscopic properties of interfacial film, which can be illustrated by its interfacial dilatational viscoelasticity investigated under different amplitude and frequencies, are of great significance to explain the mechanism of emulsion stability and demulsification, to understand the adsorption of surfactants onto droplet interface and the interaction between surfactants. Based on these investigation, the effects of surfactants structure on the emulsion stability are consequently elucidated and clarified.

**Figure 1:**
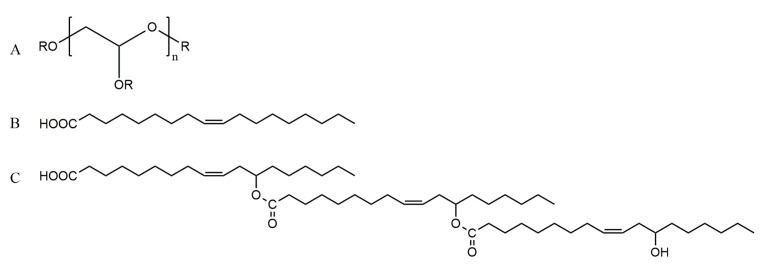
Chemical structure of polyglycerol (**A**); oleic acid (**B**) and polyricinoleate (**C**).

Polyglycerol polyricinoleate (PGPR), obtained by esterification of polyglycerol and polyricinic acid, is a polymeric and bulkier semi-synthetic lipophilic surfactant, widely used to stabilize W/O emulsions. To meet consumers’ growing needs for health care, the search for natural ingredients which may completely or partially replace PGPR is becoming a new research focus [20]. Partial replacement of PGPR were studied using lecithin and the effects on the properties of W/O emulsion were investigated [21], and using inorganic nanoparticles as emulsifying agents to obtain stable Pickering emulsions was another solution [22]. Above all, analyzing the relationship between structure and emulsifying properties of PGPR is the basis to successfully design and develop the alternatives. Therefore, W/O emulsion was constructed with PGPR and polyglycerol-2 dioleate to illustrate the structure-activity relationship of surfactant. Both of them take polyglycerol as hydrophilic head group, while their structural difference is the hydrophobic chain. The hydrophobic chain of PGPR is a polyricinoleic acid [23] synthesized by the condensation of ricinoleic acid, whereas the hydrophobic chain of diglyceryl dioleate is oleic acid only (Figure 1). Ricinoleic acid has a hydroxyl group which is absent in oleic acid, however, there are many branched chains formed onto the hydrophobic chain of polyricinoleic acid through condensation, and the length of the longer carbon chain is several times to that of oleic acid. It is conceivable that the branched chains and the longer hydrophobic chain of PGPR should significantly affect the micro-properties of interfacial film formed at the water-oil interface, and induce cascading effects onto the emulsion stability. The aim of this work was to study the effect of surfactant molecular structure on the properties of interfacial film through the investigation of dynamic interfacial tension of surfactants and interfacial dilatational viscoelasticity of the interfacial film based on amplitude sweep and frequency sweep. The relationship between surfactant molecular structure and emulsion stability was further studied to provide experimental reference and theoretical guidance for designing surfactant structure or improving surfactant properties.

## 2. Materials and Methods

### 2.1. Materials

Caprylic/capric triglyceride (ODO) was purchased from Zhengtong Chemical Co., Ltd. (Zhengzhou, China). PGPR (HLB: 1.5–2; complex with minimum 75% n-glycerols with n = 2, 3, and 4; maximum 10% m-glycerols with m ≥ 7) was purchased from Danisco Co., Ltd. (Suzhou, China). Polyglycerol-2 dioleate (HLB: 3–4, MW: 695.0 g/mol, purity ≥ 99%) was purchased from KarmaChem (Shanghai, China). Fluorescein–Sodium was purchased from Sinopharm Chemical Reagent Co., Ltd. (Shanghai, China).

### 2.2. Emulsions Preparation

W/O emulsions were prepared by magnetic stirring and high-speed shearing, respectively. In order to avoid interference of α-gel [24] to the emulsions stability and make sure the emulsions were mainly stabilized by the adsorption of surfactant to the water-oil interface, the addition of surfactant was found to be 3% of the mass of ODO. In a 50 mL beaker, 18 g of ODO containing 3% PGPR or polyglycerol-2 dioleate were mixed by magnetic stirring at a speed of 1600 rpm. The magnetic-stirring W/O emulsions were prepared by adding 6 g aqueous phase containing 10 mg/L sodium fluorescein to the oil mixture and stirring continuously for 5 min. The high-speed-shearing emulsions were obtained from magnetic-stirring emulsions after high-speed shearing at 6000 rpm for 1 min. The W/O emulsions were added into 5 mL glass vials to observe the storage stability.

### 2.3. Emulsions Microstructure

The microstructure of aqueous phase in the emulsions were observed by confocal laser scanning microscopy (CLSM, Leica Microsystems, Inc., Heidelberg, Germany). The microstructure of fresh prepared W/O emulsions were dropped on slides, sealed with cover slides, and observed with a 20× objective lens and an Ar/Kr laser with an excitation line of 488 nm. All observation were finished in 30 min.

### 2.4. Interfacial Tension-Area (π-A) Isotherm

A Teflon Langmuir trough (908 mm × 370 mm, (KSV Instruments, Helsinki, Finland) with two Teflon barriers was used to characterize the properties of surfactants. Before each measurement, the trough and barrier were thoroughly cleaned with detergent, ethanol, and deionized water. The interfacial π-A isotherm of PGPR and polyglycerol-2 dioleate at the water-oil interface were performed in a Langmuir trough with Wilhelmy-type film balance (KSV Instruments, Helsinki, Finland). When the surfactant was present at the interface, the interfacial tension (π) was calculated from the interfacial pressure without the surfactant (σ_0_) and the real-time interfacial pressure (σ) as describe by Equation (1):

(1) $$\pi =\sigma_{0}-\sigma$$

The trough was then filled with 400 mL deionized water and was swept clean by vacuum device. Then, 200 mL ODO oil solution containing 1% surfactant was carefully added as the upper phase.

Barriers were used to slowly compress the surface film at a rate of 10 mm/min while measuring the interface pressure as a function of area until the interface area came to 200 cm^2^. The barriers were then opened at the same speed until the interface area came to 400 cm^2^ and the interface pressure was also recorded. This isotherm was repeated 15 times. The water-oil interface pressure without surfactant was measured to be 21.7 mN/m.

### 2.5. Interfacial Dilatational Rheology

Dynamic viscoelastic modulus is defined as the ratio of the change in interfacial tension γ to the change in interface area (A), which can be separated into two components, i.e., elastic modulus $E_{d}$ and viscous modulus$\;E_{\eta }$ as described in Equation (2):

(2) $$\pi =\sigma_{0}-\sigma$$

For the viscoelastic interfacial films, when the interfacial area and interfacial tension change periodically with the periodical expansion and compression of the interface, there is a certain phase angle *θ* between the periodic change of interface tension and the periodic change of interface area. The $E_{d}$ and $E_{\eta }$ can be calculate by the absolute value of dilatational modulus and phase angle as described in Equations (3) and (4):

(3) $$E_{d}=\left|E\right|cos\theta$$

(4) $$E_{\eta }=\left|E\right|sin\theta$$

The interfacial dilatation rheological properties of interfacial film can be obtained by changing the amplitude and frequency during interfacial dilatation rheological experiment.

#### 2.5.1. Amplitude Sweep

The barriers were moved to the position where interface area was 140 cm^2^, the oil solution containing 1% (*w*/*w*) surfactant was added. The barriers were held for 60 min until the surfactant adsorbed completely to the oil-water interface. The linear viscoelastic region (LVR) was determined by the amplitude sweep, amplitude was varied from 1% to 28% of the interface area. The oscillation frequency was set to 10 mHz as Rühs [25] described, and the amplitude sweep was executed from small to large amplitudes, each amplitude ran for 10 sinusoidal oscillation periods, and between two oscillations followed by a time corresponding to 30 cycles without any oscillation [21], making sure the interfacial film have enough time to recover. The amplitude sweep data were analyzed by calculating the interface dilatational modulus and drawing Lissajous curves.

The interface dilatational modulus was calculated basing on the assumption that the sinusoidal strain would produce a pure sinusoidal stress response. If the strain exceeds the linear viscoelastic range, the stress response will not be a perfect sinusoidal curve, at this point, dilatational modulus might with deviation. Lissajous curve is redrawn according to the relation between the interfacial tension (δπ) and relative strain (*δa*/a_0_), where δπ is the difference between the interfacial tension and the initial interfacial tension, *δa* is the difference between the interfacial area and the initial interfacial area a_0_, and a_0_ is the initial interfacial area. Lissajous curve [18,26,27] can be used to quantify the non-sinusoidal strain response and to analyze the interfacial dilatation rheological properties of the interfacial film outside the linear viscoelastic region.

#### 2.5.2. Frequency Sweep

According to the results of amplitude sweep, the amplitude was set as 2% of interface area, and the surfactant was fully adsorbed at the oil-water interface for 60 min, the interface area was 140 cm^2^ and the frequency range was 5 to 45 mHz.

### 2.6. Emulsion Stability

Emulsion stability was determined by measuring backscattering (BS) in a Turbiscan Lab (Formulaction, Toulouse, France). Emulsion samples were placed without dilution in the test cells. Backscattered light was monitored as a function of time and cell height for 3 h at 30 °C.

### 2.7. Statistical Analyses

The dilatational elastic modulus ($E_{d}$) and dilatational viscosity modulus ($E_{\eta }$) of interfacial film were analyzed by KSV NIMA oscillatory Barrier Analysis (Version 3.80, KSV Instruments, Helsinki, Finland). ImageJ analysis software was used to determine the emulsion droplet size and distribution from the CLSM images, and the number of droplets measured per sample was larger than 200. All experiments were repeated at least twice.

## 3. Results and Discussion

### 3.1. Interfacial Tension-Area (π-A) Isotherm of Emulsifier

**Figure 2:**
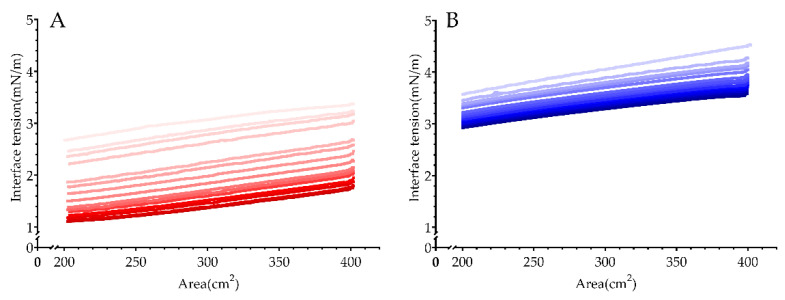
Interfacial tension-area (π-A) isotherm of surfactants at oil-water interface. (**A**) polyglycerol-2 dioleate; (**B**) PGPR (color from light to deep for 1–15 times compression).

An ideal surfactant requires to efficiently reduce interfacial tension, quickly absorb onto the interface, and fully cover the water-oil interface [8]. These properties are essentially important to the initial morphology of emulsions, such as the average diameter and distribution of droplets, as well as the emulsion stability. Figure 2 showed the interfacial tension-area (π-A) isotherms of PGPR and polyglycerol-2 dioleate after 15 cycles of compression-expansion on the oil-water interface, and both of them reduced the interfacial tension. After six cycles, the adsorption isotherm of PGPR tended to be invariable, while that of polyglycerol-2 dioleate was unstable until nine cycles. The interfacial tension of PGPR was stable at ~2.9 mN/m, and that of polyglycerol-2 dioleate decreased to 1.1 mN/m. The arrangement of two surfactants at the interface might cause the difference. Polyglycerol-2 dioleate molecules occupy smaller average molecular areas with the head group adsorbing onto the interface and the hydrophobic oleic acid chain extending into the oil phase. More polyglycerol-2 dioleate molecules were able to adsorb onto the interface when they adjusted the orientation of hydrophobic chain to reduce the average molecular area during the compression-expansion cycles, and the interfacial tension consequently decreased. The hydrophobic chain of PGPR contains many hydrophilic acyl groups and thus they can adsorb onto the water-oil interface besides the polyglycerol head group, while other hydrophobic fatty acid chains extend to the oil phase to construct spider-like conformation [28] and thus larger average molecular area is needed. Due to the large molecular steric hindrance of PGPR, saturated adsorption was achieved after only six cycles of compression-expansion and then no more PGPR molecules could adsorbed onto the interface.

The smaller interfacial tension promotes the formation of smaller droplets [29]. Therefore, the emulsion prepared by polyglycerol-2 dioleate was theoretically with smaller droplet size. The quick adsorption equilibrium PGPR reached during the compression-expansion cycles suggested that PGPR could quickly adsorb onto the droplets surface [30] during the emulsion preparation and provide a protective layer to prevent the droplets coalescence. Day [31] reported that the rapid adsorption of surfactant molecules onto the interface caused more significant effect on the emulsion stability than the decrease of interfacial tension. Therefore, the better stability could be predicted for the interfacial film formed by PGPR.

### 3.2. Interfacial Dilatational Rheological Properties of Surfactant

In general, surfactant molecules adsorb onto the oil-water interface form a viscoelastic interface film to resist the coalescence of emulsion droplets. As reported, the long-term stability of emulsions was critically affected by the strength of interfacial film rather than the interfacial tension [32]. The dilatational elasticity of interfacial film efficiently reduces the drainage rate between two droplets and resists the mechanical disturbance when two droplets flocculated [8]. Therefore, the amplitude and frequency sweeps were executed to investigate the effect of surfactant molecular structure on the interfacial film properties via interfacial dilatational rheology.

#### 3.2.1. Amplitude Sweep

**Figure 3:**
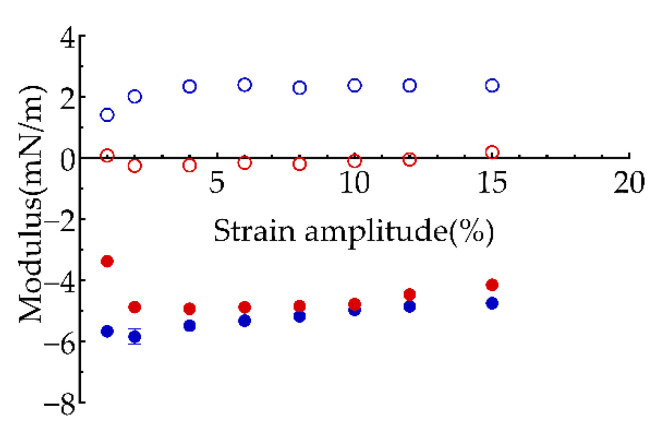
Effect of amplitude on the interface dilatational modulus of polyglycerol-2 dioleate (red) and PGPR (blue). Full and open symbols: dilatational elastic modulus and dilatational viscous modulus.

The variation of interfacial viscoelasticity of PGPR and polyglycerol-2 dioleate adsorbed onto the water-oil interface dependent on the amplitude was shown in Figure 3. The interfacial dilatational elastic modulus of the two surfactants increased firstly and decreased subsequently with the increase of amplitude, moreover, the absolute value of interfacial dilatational modulus of PGPR (blue full symbol in Figure 3) was larger than that of polyglycerol-2 dioleate (red full symbol in Figure 3). In addition, PGPR showed higher interfacial viscosity (blue open symbol in Figure 3), while polyglycerol-2 dioleate showed almost no interfacial viscosity (red open symbol in Figure 3). The low interfacial dilatational viscoelasticity under small amplitude (1%) might be limited by the measuring accuracy of instrument. The decrease of interfacial dilatational viscoelasticity with the increase of strain amplitude was due to the damage of interfacial film caused by the large amplitude. The higher interfacial viscoelasticity of PGPR was attributed to the branched chains on the hydrophobic long chain, resulting in the formation of a disordered entangled chain-like layer between the PGPR molecules at the interface [33] and enhancing their inter-molecular interactions to increase the maximum value of dilatational viscoelastic modulus [34].

**Figure 4:**
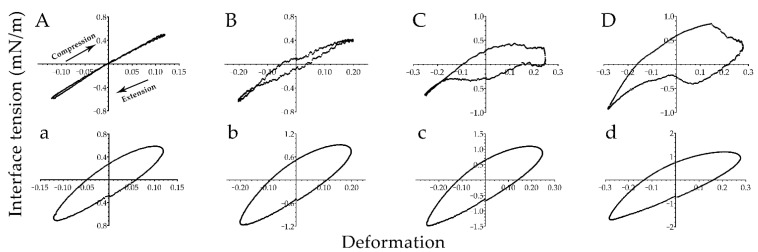
Lissajous plots obtained during amplitude sweeps (15, 20, 25, 28%) of oil-water interface stabilized by polyglycerol-2 dioleate (**A**–**D**) and PGPR (**a**–**d**), respectively.

The viscoelastic properties of interface film can be obtained by the shape of Lissajous curve, which appears a straight line for the pure elastic interface, and an ellipse for that of the viscoelastic interface [26]. Furthermore, the Lissajous curve can explain the dependence of interfacial rheological response on the interfacial microstructure. Van [27] studied the rheological response of air-water interface stabilized by oligofructose fatty acid esters. The interfacial rheological response was attributed to the 2D soft glass phase structure formed by the ester, which was strain-softening upon expansion and strain-hardening upon compression. As shown in Figure 4, the Lissajous curve of PGPR was ellipse at 15% amplitude indicating that the PGPR interface was of certain viscoelasticity, while the Lissajous curve of polyglycerol-2 dioleate was a straight line demonstrating that the polyglycerol-2 dioleate interface exhibited elasticity rather than viscosity. The results were consistent with those of amplitude sweep (Figure 3). Furthermore, the curves’ slope of PGPR increased with the increase of amplitude upon compression, which points to strain hardening. In contrast, the curves’ slope decreased with the increase of amplitude upon extension, which points to strain softening. For polyglycerol-2 dioleate, the similar observation of the strain-hardening in compression and strain-softening in expansion was also recorded in the Lissajous curves at higher amplitude, however, the curves were more irregular. This was on account of the smaller interfacial viscosity of polyglycerol-2 dioleate which leaded to the failure of interfacial film to resist the disturbance caused by compression and expansion. The coalescence of two perfectly monodisperse droplets results in a 20% change of emulsion surface area if the total volume remains constant [35]. Therefore, to produce similar disturbance during droplet coalescence, three amplitudes gradients of 20%, 25% and 28% was applied to investigate the properties of interface film. The results showed that the interfacial viscosity of PGPR effectively reduced the influence of interfacial area change on the interfacial structure assembled via surfactants. On the contrary, for polyglycerol-2 dioleate, the lack of interfacial viscosity seriously broke the interfacial structure, disturbing the integrity of interfacial film at high amplitudes. The different resistance of interfacial film formed by the two surfactants could be induced by the disordered entangled chain layer of PGPR molecules which could avoid the damage of interfacial film by changing the conformation of molecular chain under strain. However, the interfacial structure of polyglycerol-2 dioleate was destroyed if raised the strain since the interfacial area of polyglycerol-2 dioleate only responded to the interfacial area change by compact packing.

#### 3.2.2. Frequency Sweeps

The variation of interface dilatational modulus with the oscillation frequency could reflect the disturbing of interface. The amplitude of 2% was applied in this study according to the amplitude sweep result to ensure that the amplitude of frequency sweep unchanged the interfacial structure, and the frequency range of 5–45 mHz was used to simulate the external disturbance at low frequency of interface film. The dilation modulus change of interface film with the test frequency was controlled by the interface relaxation. For the surfactant with lower molecular weight, two types of relaxation are generally suggested. One is the diffusion of molecules from the bulk phase to the interface, and the other is the relaxation of molecules at the interface, such as molecular orientation, molecular rearrangement, and so on [36]. At present, the data of interface dilatational modulus in most cases is interpreted with diffusion relaxation [37]. The diffusion relaxation time between the interface and the bulk phase was long enough at lower oscillation frequency, and the lower dilatational modulus was characterized. However, at higher oscillation frequency, the shorter detection duration limited the diffusion relaxation of interfacial film and the interfacial film dilatational modulus was consequently higher.

**Figure 5:**
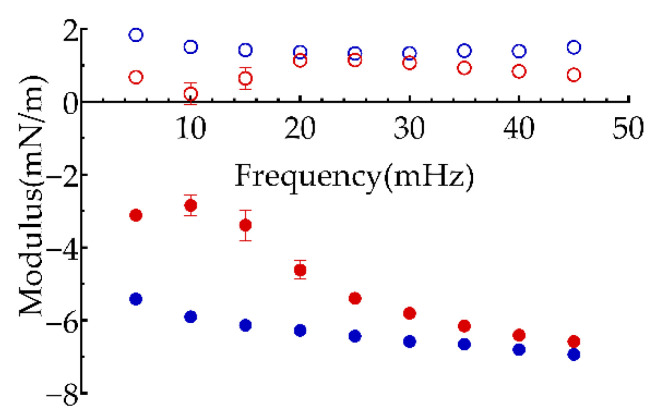
Effect of oscillation frequency on the viscoelasticity of interfacial film formed by polyglycerol-2 dioleate (red) and PGPR (blue). Full and open symbols: dilatational elastic modulus and dilatational viscous modulus.

The frequency sweeps (Figure 5) showed that the elasticity modulus of two surfactants was larger than the viscosity modulus in the detected range of oscillation frequency, suggesting that the surfactants were more elastic at the interface. With the frequency increased, the dilatational elastic modulus of PGPR increased slightly, while its dilatational viscosity modulus stabled at 2 mN/m. Both dilatational elastic modulus and dilatational viscos modulus of polyglycerol-2 dioleate were lower at low oscillation frequency, which gradually increased to similar values to those of PGPR when frequency increased.

The significant change in the dilatational elasticity modulus of polyglycerol-2 dioleate in the range of oscillation frequency was observed. The interfacial viscoelastic modulus was reported mainly affected by the density of surfactant molecules at the interface [9,38]. When the interface expanded, the density of surfactant molecules at the interface decreased, improving the interface tension. At the same time, molecules from the bulk phase would spread to the newly formed interface to eliminate the interfacial tension gradient. The straight hydrophobic chain of polyglycerol-2 dioleate hydrophobic chain only brought weak interaction between the hydrophobic chains, thus, it could diffuse during low-frequency oscillation since the energetic barrier overcoming interfacial and bulk phase diffusion was quite low. What’s more, the relatively low molar mass might promote the diffusion [39]. Thus it reduced the change of interface tension and increased the density, and the viscoelastic modulus generally declined. For PGPR, the disordered entangled chain-like structure was formed via the hydrophobic chains of PGPR built stronger interaction among them, and the acyl groups on PGPR could be embedded in the oil-water interface [28] which produced higher energetic barrier when desorbing from the interface, which hindered the diffusion between the phase and the interface and led to a larger change of interface tension and density, thus higher dilatational elasticity modulus was observed during the whole detected frequency range. Moreover, the lower interfacial dilatational modulus of polyglycerol-2 dioleate at low frequency might further led to the instability of interfacial film during storage, resulting in the macro-phase separation of emulsion.

### 3.3. Emulsion Stability

This study was aimed to discuss the influence of surfactant molecular structure on the emulsion stability, so the emulsion was only prepared by relatively low-energy procedures, i.e., magnetic stirring and high-speed shearing. Compared with the emulsion prepared through high-energy procedures like high pressure homogenization, the size distribution of prepared emulsion in our study was larger, and the emulsion instability could be observed within several days, which was beneficial to detect the influence of surfactants on the stability of emulsion interfacial film and emulsion. Emulsion stability could be evaluated by calculating the t25 as Atanase [9] did for emulsions destabilize in few hours, or be determined using LUMiSizer [40] to accelerate sedimentation by centrifugation. Turbiscan Lab [41] gives kinetic information on the process leading to phase separation. Thus, it was applied in this work.

The stability of W/O emulsions prepared by two surfactants were significantly different as the backscattering profile shown. The destabilization of emulsions prepared by polyglycerol-2 dioleate was dominated by coalescence in the initial period, which was evidenced by a progressive decrease in backscattering. The coalescing droplets grew rapidly and led to the phase separation. For the two emulsions, the one prepared by magnetic stirring destabilized faster. Emulsions stabilized by PGPR underwent totally different destabilization process. The magnetic-stirring emulsion coalesced at first, as the coalescing droplets became bigger, then they migrated from the top to the bottom of the emulsion, causing a fall in backscattering at the sample top due to the clarification of emulsion as droplets fallen down and an increase in backscattering at the sample bottom due to the increase of droplets concentration as sediment formed. The high-speed-shearing emulsion was more stable, and the coalescence of emulsion during the test period due to an insignificant sedimentation.

**Figure 6:**
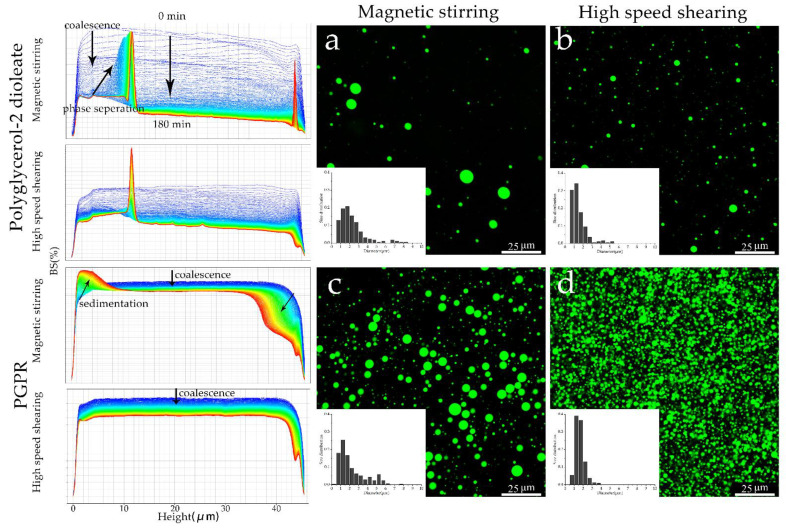
Changes of emulsion stability during preparation and storage (**left**); CLSM images of fresh prepared emulsions, a-d corresponding to the emulsions prepared by magnetic stirring and high speed shearing using polyglycerol-2 dioleate and PGPR as emulsifier respectively (**right**).

The droplet size and distribution of fresh prepared emulsion were analyzed based on their CLSM images using ImageJ. The particle sizes of emulsions emulsified with polyglycerol-2 dioleate were 2.1 ± 1.6 μm prepared by magnetic stirring and 1.2 ± 0.8 μm prepared by high-speed shearing, respectively. If emulsified with PGPR, the corresponding sizes were 1.7 ± 1.4 μm and 1.5 ± 0.6 μm, respectively. Apparently, the droplets prepared by two surfactants were similar in size and size distributions if under the same preparation procedure, meanwhile, smaller and more uniform droplets were achieved in the emulsion undergoing high-speed shear. The results showed that the two surfactants could help the inner water phase well-dispersed during the preparation of emulsion. However, the rapid destabilization of emulsions prepared by polyglycerol-2 dioleate were quickly observed, which was also clearly showed in CLSM observation that there were fewer droplets dispersed comparing with the emulsion prepared by PGPR. As shown in Figure 6c, the droplets were fewer than that in Figure 6d and more than that in Figure 6a, it means emulsions using PGPR as surfactant showed better stability and it was improved by high speed shearing.

### 3.4. Influence of Surfactant Molecular Structure on Emulsion Stability

The emulsions with well-dispersed inner aqueous phase were successfully prepared by both PGPR and polyglycerol-2 dioleate, however, the emulsion produced by the latter rapidly destabilized, which was mainly related to the flocculation, coalescence and Ostwald ripening of emulsion during preparation and storage and affected by the difference of molecular structure.

Emulsion coalescence refers to the coalescence of two droplets into a single droplet and relates to the thinning and rupture of liquid film between droplets, which is intensively influenced by the interfacial properties of adsorption layer. Coalescence in emulsion may occur as a result of collision between moving droplets or flocculation. The rapid adsorption of PGPR at the interface favored the resistance of coalescence between the newly formed droplets and prevented the rapid destabilization of emulsion (Figure 6). The interfacial rheological properties of interfacial film were particularly important when discussing the rupture of liquid film or coalescence of droplets from the view of fluid mechanics. The dilatational viscoelasticity was of incredible importance, to be more specific, the high interfacial dilatational viscoelasticity effectively reduced the coalescence between flocculating droplets to resist the deformation of liquid interfacial film responded to the mechanical disturbance. The higher stability of emulsion prepared from PGPR was attributed to the higher interfacial dilatation viscoelasticity of interfacial film assembled by PGPR which obtained stronger resistance to interfacial disturbance during amplitude sweeps. The emulsion Ostwald ripening is a partial dissolution of dispersed liquid phase caused by capillary pressure, resulting in mass transfer from small droplets to large droplets. Although it is mainly driven by the solubility of dispersed liquid, it is also influenced by the presence of surfactants and the rheological properties of interfacial film. During Ostwald ripening, the slow compression-expansion rate of interface was recorded. The deformation of interface film during Ostwald ripening was studied by low frequency oscillation, in which the diffusion of polyglycerol-2 dioleate between the interface and the bulk phase decreased the interfacial dilatational viscoelasticity, and consequently the emulsion was destabilized if Ostwald ripening occurred.

The adsorption rate of surfactants at the interface and their dilatational rheological properties varied mainly attributing to the difference of the hydrophobic chain structure of the two emulsifiers. The branched chains grafted on the hydrophobic chain of PGPR provided higher intermolecular interaction at the water-oil interface, at the meanwhile, more acyl groups in the molecule embedded in the interface prevented the diffusion of surfactants to generate faster adsorption rate and higher interfacial dilatation viscoelasticity of the interfacial film.

## 4. Conclusions

This work investigated the effect of different hydrophobic chain structure in PGPR and polyglycerol-2 dioleate on the interfacial film properties based the surface dilatational rheology, and the cascading effect on the emulsion stability was also discussed. The results indicated the branched hydrophobic chains of PGPR, compared with the straight hydrophobic chain on polyglycerol-2 dioleate, was critically important to affect the interfacial adsorption rate and the interfacial dilatational viscoelasticity. PGPR quickly adsorbed onto the water-oil interface during emulsification to maintain the stability of newly formed emulsion droplets, and its higher dilatational viscoelasticity helped the emulsion resist the disturbance of interfacial film caused by coalescence and Ostwald ripening during storage and exhibited higher emulsion stability. This study reveals profound understanding of the influence of surfactant molecular structure on the interfacial tension and interfacial viscoelasticity and their cascading effects on the emulsion stability, providing experimental reference and theoretical guidance for the design and selection of proper surfactants.

## Data Availability

The data presented in this study is available on request from the corresponding author.
